# Greater Mouse-Eared Bats (*Myotis myotis*) Hibernating in the Nietoperek Bat Reserve (Poland) as a Vector of Airborne Culturable Fungi

**DOI:** 10.3390/biology10070593

**Published:** 2021-06-27

**Authors:** Justyna Borzęcka, Agata Piecuch, Tomasz Kokurewicz, Kathleen H. Lavoie, Rafał Ogórek

**Affiliations:** 1 Department of Mycology and Genetics, University of Wrocław, Przybyszewskiego Street 63-77, 51-148 Wrocław, Poland; agata.piecuch@uwr.edu.pl; 2 Department of Vertebrate Ecology and Paleontology, Institute of Environmental Biology, Wrocław University of Environmental and Life Sciences, Kożuchowska 5b, 51-631 Wrocław, Poland; tomasz.kokurewicz@upwr.edu.pl; 3 Department of Biological Sciences, State University of New York, Plattsburgh, NY 12901, USA; lavoiekh@plattsburgh.edu

**Keywords:** aeromycota, *Myotis myotis*, Nietoperek bat reserve, air quality

## Abstract

**Simple Summary:**

Fungi and bats are important parts of many ecosystems where they play critical ecological roles. Bats can contribute to an increase of airborne fungi in underground ecosystems, which may cause allergies and infections in mammals. Our study contributes to gaining new knowledge about aeromycota present in the close vicinity of the hibernating greater mouse-eared bat (*Myotis myotis*) in an underground site. *M. myotis* is the most common bat species in Europe with direct human contact. Hibernating *M. myotis* contribute to an increase in the concentration of aeromycota in the underground site and is a vector/reservoir of microscopic fungi, including those that are potential threats to mammal populations; however, the concentration of aeromycota at this site does not pose a threat to human health.

**Abstract:**

Bats can contribute to an increase of aeromycota in underground ecosystems and might be a vector/reservoir of microorganisms; however, there is no information about the number and species composition of fungi around hibernating bats. One of the most common species in Europe with direct human contact is the greater mouse-eared bat (*Myotis myotis*). The goal of our research was the first report of the airborne fungi present in the close vicinity of hibernating *M. myotis* in the Nietoperek bat reserve (Western Poland) by the use of culture-based techniques and genetic and phenotypic identifications. Aerobiological investigations of mycobiota under hibernating bats were performed on two culture media (PDA and YPG) and at two incubation temperatures (7 and 24 ± 0.5 °C). Overall, we detected 32 fungal species from three phyla (*Ascomycota*, *Basidiomycota*, and *Zygomycota*) and 12 genera. The application of YPG medium and the higher incubation temperature showed higher numbers of isolated fungal species and CFU. *Penicillium* spp. were dominant in the study, with spores found outside the underground hibernation site from 51.9% to 86.3% and from 56.7% to 100% inside the bat reserve. *Penicillium chrysogenum* was the most frequently isolated species, then *Absidia glauca*, *Aspergillus fumigatus*, *A. tubingensis*, *Mortierella polycephala*, *Naganishia diffluens*, and *Rhodotorula mucilaginosa*. Temperature, relative humidity, and the abundance of bats correlated positively with the concentration of airborne fungal propagules, between fungal species diversity, and the concentration of aeromycota, but the number of fungal species did not positively correlate with the number of bats. The air in the underground site was more contaminated by fungi than the air outside; however, the concentration of aeromycota does not pose a threat for human health. Nevertheless, hibernating bats contribute to an increase in the aeromycota and as a vector/reservoir of microscopic fungi, including those that may cause allergies and infections in mammals, and should be monitored.

## 1. Introduction

Bats (Chiroptera) are one of the most versatile placental mammals. They can be found in almost every part of the world, except Antarctica [1]. These nocturnal animals have always interacted with humans and were often used in traditional medicine or as food since Prehistoric times by some indigenous tribes living in the Asian-Pacific region [2]. Worldwide, bats are an important part of many ecosystems, where they play critical ecological roles. Bat guano is rich in nitrates and has been, and sometimes still is, mined from caves to be used as fertilizer. Moreover, guano deposits in caves are a major source of nutrients for guanobitic cave fauna [3]. The majority of bat species are insectivorous; thus, they control agricultural pest populations and reduce the need for pesticides [4]; they are involved in the reduction of the populations of some insects that are harmful for humans, such as mosquitoes (Culicidae family) and biting midges (Simuliidae and Ceratopogonidae families); they are plant pollinators; and aid in seed dispersal, maintaining tree diversity, and supporting habitat regeneration [5]. Some species, like the greater mouse-eared bat, *Myotis myotis*, can also serve as a tourist attraction, since they annually change their roosts, often inhabiting human settlements such as loft spaces and attics and forming summer nursery colonies [6]. In many places in the United States, flying bats are an attraction for tourists. Such is the case of Carlsbad Cavern and the Congress Street Bridge in Austin, Texas [7,8]. However, it is believed that bats can also have a negative impact on human health and act as a natural reservoir of many zoonotic pathogens, such as viruses, bacteria, or fungi [9,10,11,12]. Due to their high mobility and a complex social life, bats can easily spread diseases among themselves, as well as transfer them to humans, posing a potential threat to all of us [12,13].

Temperate zone bats have a well-defined annual cycle, largely determined by climate. *Myotis myotis* performs annual dispersions, choosing human localities as their maternity roosts and inhabiting low-temperature zones as hibernacula during winter. Before the hibernation starts, these animals cluster together in caves or underground sites and fly in a circular motion, described as swarming, that precedes mating. Before the winter starts, *M. myotis* gather in large groups [14]. Both of these behaviors create an excellent opportunity to spread infectious agents, such as fungi, viruses, and/or bacteria, among individuals [15,16,17]. Bat hibernation sites are crucial to survive harsh seasonal environmental conditions, such as reduced insect availability, and are characterized by low temperatures (around 2 to 10 °C) and high relative humidity, often above 80%, allowing these animals to minimize energy expenditure [18].

The majority of infectious agents are not able to actively grow in underground ecosystems due to the low temperatures, no light, and scarcity of nutrients [16,19]. Underground sites are inhospitable places for nonresidential microbial life [20]. Although the majority of fungi can grow at 20 to 25 °C, they should be able to survive and grow at 36 to 37 °C to be considered human pathogens. It is clear that the resident mycobiota found underground will mostly comprise psychrotolerant and psychrophilic fungi [21,22,23,24], such as *Pseudogymnoascus destructans* (Pd), causing white-nose syndrome (WNS) in bats and having optimal growth between 12.5 and 15.8 °C [25,26,27]. The virulence of Pd is also due its preference for nitrogenous substrates occurring in bat skin and lipids [28]. Organic debris such as bat guano, plant material, and animal carcasses and wastes are crucial substrates for the development and growth of underground fungi [29,30,31]. Moreover, many microorganisms, as well as other artifacts such as cellular fragments, fungal spores, or various mycotoxins and enzymes, can be found in an aerosol form [32]. These fungi enter caves from external environments mainly due to air currents; water from floods or surface seeps; or carried in by humans, animals, and arthropods [15,33,34,35]. However, three questions arise: How do some fungi, including pathogens, survive in such conditions? How do they get there? What airborne fungi are associated with *M. myotis* in underground sites?

Most previous studies were focused on the examination of hibernating bats’ wing membranes, which indeed are rich in fungal species, especially *Ascomycota* [10,21,36]. The skin fungal assemblages of bats vary based on their susceptibility to white-nose syndrome [37]. Unfortunately, there is still a lack of comprehensive data on airborne fungi related to specific species of bats, with only two reports published about bat hibernation sites in relation to airborne fungi [10,15] and one report about a bat hibernation site in relation to airborne bacteria [16]. These first two studies showed that bats are the main vector/reservoir of airborne fungi such as *Aspergillus* and *Penicillium* species in underground ecosystems in Western Poland and Northeastern Brazil [10,15]. *Aspergillus* and *Penicillium* can pose a serious threat for both animals and humans due to an extensive production of spores and mycotoxins, resulting in allergies, mycosis, mycotoxicosis, and even systemic infections [20,38]. Thus, the microbiological monitoring and control of bats’ health could play a significant role in the protection of both humans and bats, especially since many bat species are listed as endangered in the Red List of the International Union for Conservation of Nature (IUCN), and some are threatened or near-threatened [39]. *Myotis myotis* is in the IUCN status of least concern, but there have been recent population declines in some areas, and it is extirpated in some places in Northwestern Europe. For these reasons, the species is listed in Annex II of the European Union Habitat Directive and requires special protection measures [40].

The main goal of our research was to investigate the airborne culturable fungi associated with hibernating greater mouse-eared bats (*Myotis myotis*) in the Nietoperek underground by determining the number and species composition of the aeromycota to analyze: (1) the relationship between the fungal species composition and the number of bats, (2) the influence of air temperature and humidity on the number and species composition of airborne culturable fungi, (3) the influence of the culture medium and incubation temperature on the concentration and species composition of fungal spores in the air, and (4) whether the mycological quality of air within this underground poses a risk to human health, especially to the tourists.

## 2. Materials and Methods

Samplings were made in the Nietoperek bat reserve under license no. WPN-I.640 l.369.2015.JK, issued by the Regional Directorate for Environmental Protection in Gorzów Wielkopolski, Poland. The study was performed on 9 January 2016 and included the counting and visual identification of bat species, measurements of the microclimatic parameters, and passage dimensions, as well as the collection of air samples for mycological analyses.

### 2.1. Study Area

**Figure 1:**
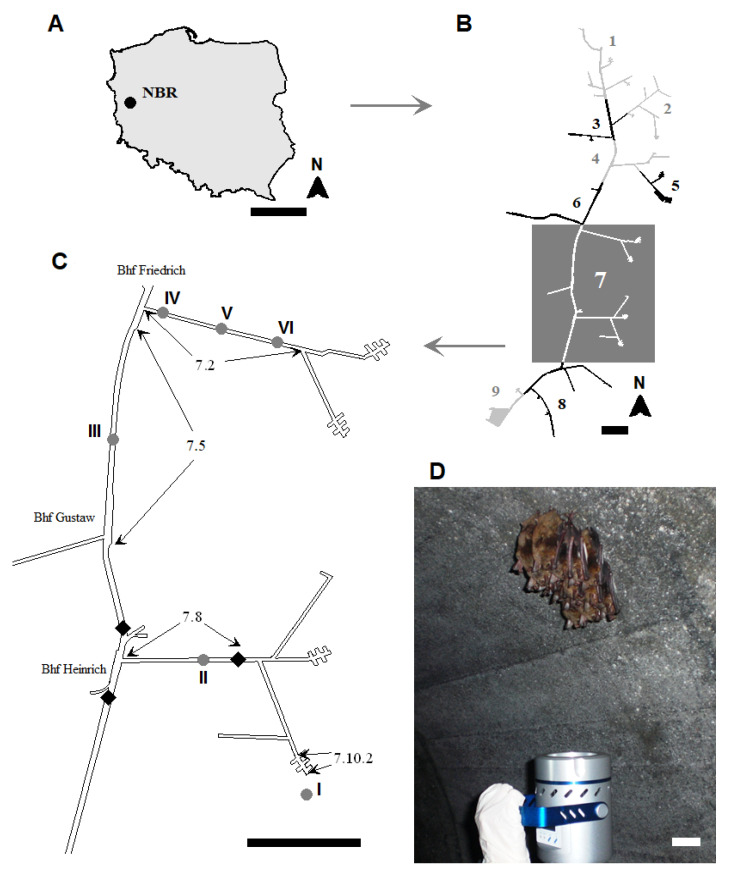
(**A**) Geographic location of Nietoperek bat reserve (NBR) in Western Poland. Scale bar = 250 km. (**B**) The outline of the underground fortification system divided into 9 sections for bat monitoring purposes. Scale bar = 500 m. (**C**) Area highlighted in (**B**) shows sampling sites I-VI in section 7 and one site outside the underground fortification system (location I—near section No. 7.10.2) and inside the underground fortification system in sector No. 7: locations II (section No. 7.8), III (section No. 7.5), IV, V, and VI (respectively, beginning of section No. 7.2, center, and end of it). Scale bar = 500 m. (**D**) Air sampling (locations from IV–VI) under hibernating *M. myotis* for the mycological analysis. Black diamonds mark security gates. Scale bar = 5 cm.

The 100-km-long Międzyrzecz Fortified Front (Figure 1), Ostwall or Festungsfront im Oder-Warthe Bogen situated in Western Poland (52°25′ N, 15°32′ E), was built by Germany in 1934–1944. Its ca. 15-km-long central sector “Wysoka” (Zentralabschnitt or Abschnitt Hochwalde) has a concentration of fortifications, including a system of concrete tunnels with a total length of ca. 32 km situated 20–30 m underground and standalone bunkers. The major part of the system has a stable microclimate, but a dynamic microclimate is found near the entrances [41,42]. There is an area open to the public for sightseeing, but no records of the amount of visitations is kept. A 1-km corridor is open year-round, but the side corridors are closed during hibernation season (15 October–15 April) to protect the large numbers of hibernating bats from disturbances.

The Nietoperek bat reserve situated in the central sector of “Wysoka” is the largest bat hibernation site in Poland and one of the ten-largest in the European Union. The maximal number of bats recorded there in January 2020 exceeded 39,658 individuals of 8 species and species group (*Myotis mystacinus*/*brandtii*/*alcathoe*) that are indistinguishable without handling. To protect the hibernating bats and their foraging areas around the fortifications, the underground system and the surrounding surface area of 7377.37 ha became protected in November 2007 as the Natura 2000 site Nietoperek (area code: PLH080003). Out of 12 bat species found hibernating in there, four are mentioned in Annex II of the European Union Habitat Directive [40] (e.g., *Barbastella barbastellus*, *M. myotis*, *M. dasycneme*, and *M. bechsteinii*). More information about Nietoperek bat reserve was given in previous publications [15,41,42].

### 2.2. Aeromycological Study

#### 2.2.1. Sampling Methods

The isolation of fungi was performed using conventional culture methods. The microbial air sampler MAS100-ECO (MBV AG, Stäfa, Switzerland) and two different culture media were used for the mycological evaluation of the air: PDA (Potato Dextrose Agar, BioMaxima, Lublin, Poland) and YPG medium (Yeast extract Peptone Glucose: 10.0-g·L^−1^ yeast extract, 20.0-g·L^−1^ peptone, 20.0-g·L^−1^ glucose, and 15.0-g·L^−1^ agar). The samples were taken from five locations inside (sections 7.2, 7.5, and 7.8) the underground tunnels of Nietoperek bat reserve and one location situated ca. 5 m in front of the entrance (near section 7.10.2) (Figure 1). The collision method was used where air was directed over culture media, and spores were collected.

The microbial air sampler was positioned at a distance of 0.7–1 m from clusters of *M. myotis* (locations IV–VI), or it was positioned 1.5 m above the level of the floor (locations I–III) (Figure 1). It was programmed for air sample volumes of 50 L and 100 L in triplicate for each volume. The incubation of samples using both culture media was carried out at 7 ± 0.5 °C and 24 ± 0.5 °C for 5 or up to 42 days in darkness (from the first to last appearances of the colonies). We used 7 °C to isolate psychrophilic and psychrotolerant fungi and 24 °C to isolate mesophilic fungi. After incubation, fungal colonies on the plates were counted and the average colony-forming units expressed as CFU per cubic meter of air. Colonies of fungi were subcultured on plates using the same medium as the original isolation and incubated in the dark at both 7 ± 0.5 °C and 24 ± 0.5 °C for 4 or up to 35 days. After incubation, fungi were purified by the single spore method and were subcultured on PDA slants for morphological and molecular identification.

#### 2.2.2. Identification of Airborne Fungi

To identify the isolated fungi, a combination of phenotypic and molecular methods was used. For this purpose, macroscopic and microscopic observations on PDA and CYA (Czapek Yeast autolysate Agar: 30.0-g·L^−1^ sucrose, 15-g·L^−1^ agar, 5.0-g·L^−1^ yeast extract, 3.0-g·L^−1^ NaNO_3_, 1.0-g·L^−1^ K_2_HPO_4_, 0.5-g·L^−1^ KCl, 0.5-g·L^−1^ MgSO_4_·7H_2_O, and 0.01-g·L^−1^ FeSO_4_·7H_2_O) were made. The observed features included colony color and growth, as well as the occurrence of specific morphological structures like spores. The observations were analyzed according to the monographs [43,44,45,46,47,48]. Additionally, the phenotypes of some fungi were also compared with the strains from R. Ogórek’s collection (Department of Mycology and Genetics, University of Wrocław, Wrocław, Poland). The ITS sequences were run through the BLASTN search page using the Megablast program (National Center for Biotechnology Information, Bethesda, MD, USA), where the identical hits and their accession numbers were obtained.

To confirm the species affiliation, the fungal rDNA ITS (internal transcribed spacer) was sequenced. DNA was isolated from fungal colonies cultured on PDA according to the original, hexadecyltrimethylammonium bromide (CTAB)-based method [49], with minor modifications [50]. Fungal rDNA was amplified using the primers ITS1 (5′-TCCGTAGGTGAACCTGCGG-3′) and ITS4 (5′-TCCTCCGCTTATTGATATGC-3′) [51]. PCR was performed in a T100 Thermal Cycler (Bio-Rad, Berkeley, CA, USA), according to Ogórek et al. [52]. The PCR products were verified by electrophoretic separation on a 1.2% agarose gel and subsequently purified using Clean-UP (A&A Biotechnology, Gdańsk, Poland) and sequenced by Macrogen Europe (Amsterdam, The Netherlands, http://dna.macrogen.com/eng/, accessed on 19 May 2017).

### 2.3. Bat Number and Species Composition

For bat monitoring purposes, the Nietoperek underground was divided into 9 sections, and our research was performed in 3 parts of section no. 7 (sections 7.2, 7.5, and 7.8), situated in the central part of the underground system (Figure 1). Bats were counted and species were determined using the identification key by Dietz and von Helversen [53].

### 2.4. Measurements of Underground Corridors, Temperature, and Relative Humidity

The measurements of the widths, heights, and lengths of the underground corridors were carried out during the fieldwork to ±1 m using the platform QGIS (Quantum Geographic Information System, QGIS 3.12.3-București). The air temperature and relative humidity (RH) were measured nine times at each sampling site (from I to VI; Figure 1) using a thermohygrometer (AB-171 data logger, Abatronic, Radom, Poland, accuracy: ±0.1 °C, ±5% RH), recorded as the mean ± standard deviation (SD).

### 2.5. Data Analyses

The PCR product sequences were analyzed using a BioEdit Sequence Alignment Editor (http://www.mbio.ncsu.edu/bioedit/bioedit.html, accessed on 15 March 2019). The fungal ITS sequences were compared with those deposited in the GenBank of the NCBI using the BLAST algorithm (http://www.ncbi.nlm.nih.gov/, accessed on 20 March 2019). Sequences were placed in GenBank databases, accessed on 26 March 2019.

The data obtained from the number of airborne fungal colonies and microclimatic parameters were analyzed using the Statistica 12.0 package (StatSoft Polska Sp. z o.o., Kraków, Poland) using a one-way analysis of variance (ANOVA) and Tukey’s HSD (honest significant difference) test at α ≤ 0.05. Prior to the ANOVA, the percentage data were transformed to Bliss [54] angular degrees by applying the formula y = arcsin (value%)^−0.5^. After transformation, the variance was approximately constant, allowing the ANOVA to compare particular components [54]. Additionally, to determine the species diversity of airborne fungi at specific study sites, the Shannon Diversity Index (H) was used. It was calculated using the following equation: H = −Σ P_i_(lnP_i_), where P_i_ stands for the proportion of each species in the sample [55]. The Pearson (r) correlation coefficient was used to determine the relation between the temperature and humidity of the air, species diversity of airborne fungi, and the concentrations of airborne fungal propagules. This correlation was also calculated to investigate the relationships between the number of bats and the number of airborne fungal propagules in the three underground sections where bats were present.

## 3. Results

This research was carried out in five locations inside the underground facility from three different sections and one location outside near the entrance to the underground (near section 7.10.2; Figure 1). Each corridor section differed in length, height, and width; therefore, section 7.8 (study site II) had 2943.6 m^3^ of volume, section 7.5 (study site III) had 12,728 m^3^, and section 7.2 (study sites from IV to VI) had 3546.4 m^3^. The measurements of the microclimatic conditions (Table 1) showed that the highest air temperature was reported in study site number III (10.50 ± 0.00 °C; *p* _study sites II, III_ = 0.004870), and the lowest air temperature was documented in the area outside the underground (8.77 ± 0.07 °C; *p* _study sites I, VI_ = 0.004870). The highest air relative humidity was recorded in study site IV (82.06 ± 0.25%; *p* _study sites IV, V_ = 0.000134) and the lowest in study site I (56.20 ± 0.24%; *p* _study sites I, II_ = 0.000135).

In total, seven different species of bats were found in the Nietoperek bat reserve corridors: namely, *Myotis myotis*, *Myotis daubentonii*, *Myotis nattereri*, *Myotis dasycneme*, *Barbastella barbastellus*, *Plecotus auritus*, and a species that has not been identified (Table 2). The most numerous species was *M. myotis*, which constituted from 73.9% to 92.6% of all the reported bats, respectively, for section 7.5 (study site no. III) and section 7.2 (study sites no. IV, V, and VI). The largest number of bats in terms of the total number and per 1 m^3^ of the corridor was recorded in study site no. III (2775 individuals overall, with a bat density of 0.218 per 1 m^3^), and the smallest number of bats was found in study site no. II (4 bats, with only 0.001 bat per 1 m^3^) (Table 2).

Aerobiological investigations of mycobiota were performed on two culture media (PDA and YPG) and at two incubation temperatures (7 and 24 ± 0.5 °C). Phenotypic and genotypic analyses of the fungal cultures obtained in this way allowed them to be classified into 32 species, which belonged to three phyla (*Ascomycota*, *Basidiomycota*, and *Zygomycota*) and 12 genera (*Absidia*, *Aspergillus*, *Botrytis*, *Cladosporium*, *Debaryomyces*, *Neoascochyta*, *Filobasidium*, *Mortierella*, *Mucor*, *Naganishia*, *Penicillium*, and *Rhodotorula*). All the fungal nucleotide sequences (ITS rDNA) were submitted to GenBank under the accession numbers from MK690542 to MK690573. Based on a BLAST analysis, the E values amounted to zero, and the percentages of the query cover and identity ranged from 94.0–100% and 92.93–100%, respectively (Table A1). The YPG medium showed higher efficacy than PDA in isolating more fungal species. Moreover, *Aspergillus fumigatus*, *Mucor flavus*, *Mucor fragilis*, *Penicillium cavernicola*, *Penicillium commune*, *Penicillium camemberti*, *Penicillium expansum*, and *Penicillium robsamsonii* were isolated only on YPG, while *Mortierella polycephala* was cultured only on PDA (Table A2 and Table A3).

**Figure 2:**
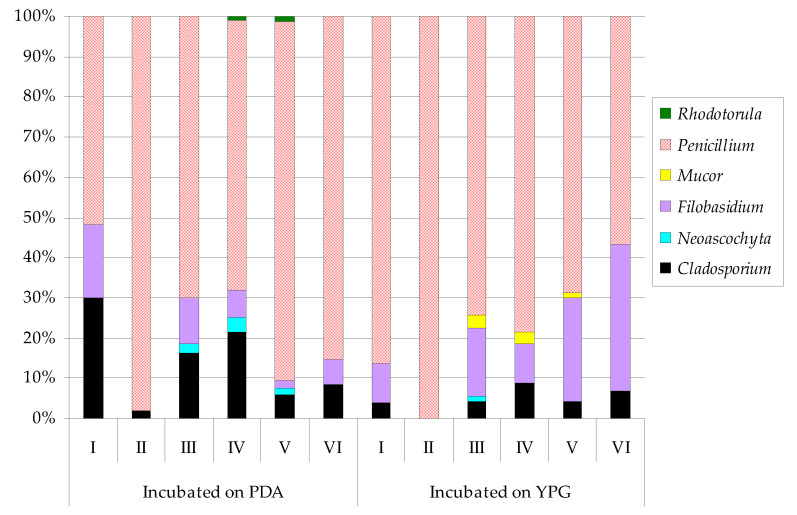
Percentage of each airborne fungal genus contributing to the total fungi cultured at 7 ± 0.5 °C from the underground hibernation Nietoperek bat reserve. Study site number I was outside the underground reserve and locations from II to VI inside it. The air sampler was positioned 1.5 m above the level of the floor at study site numbers from I to III and at a distance of 0.7–1 m from clusters of *M. myotis* in locations from IV to VI.

**Figure 3:**
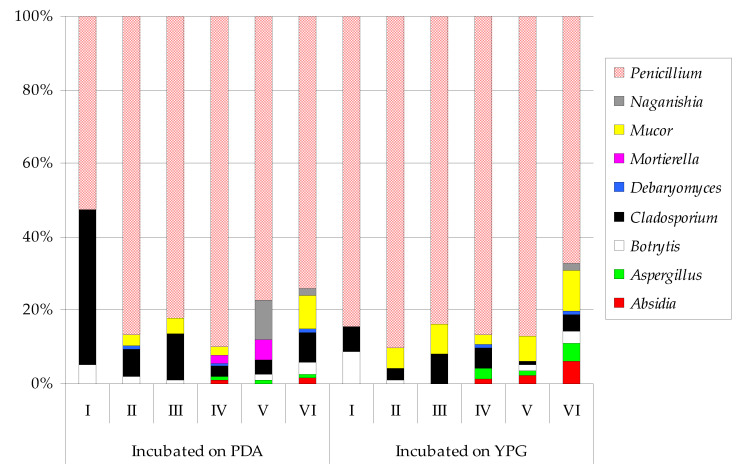
Percentage of each airborne fungal genus contributing to the total fungi cultured at 24 ± 0.5 °C in the underground hibernation sites in the Nietoperek bat reserve. Study site number I was outside the underground object and locations from II to VI inside it. The air sampler was positioned 1.5 m above the level of the floor at study site numbers from I to III, and it was positioned at a distance of 0.7–1 m from clusters of *M. myotis* in locations from IV to VI.

Overall, at the incubation temperature of 7 ± 0.5 °C, six genera of fungi were isolated from the air samples using both culture media. Of these, half of them were outside the underground site (*Cladosporium*, *Filobasidium*, and *Penicillium*) (Figure 2). At the higher incubation temperature of 24 ± 0.5 °C, the number of fungal genera increased to nine, and, similarly, only three of these nine were cultured from external air samples (*Botrytis*, *Cladosporium*, and *Penicillium*) (Figure 3). The fungi of *Penicillium* were dominant in these experiments, regardless of the type of culture medium used and the incubation temperature. The spores of *Penicillium* found outside the bat underground hibernation site (study site number I) constituted from 51.9% to 86.3% of all the captured fungal spores and were especially predominant on the YPG medium. The *Cladosporium* genus in the samples from the external location was also isolated on PDA at both plate incubation temperatures, representing 29.9% (at 7 ± 0.5 °C) and 42% (at 24 ± 0.5 °C) of all the isolated airborne fungi. In turn, the *Penicillium* genus in the air inside the underground wintering area accounted for from 56.7% to 100% of all the reported airborne fungi. *Filobasidium* was also present at the lower incubation temperature, while *Mucor* was found at the higher temperature (Figure 2 and Figure 3).

**Figure 4:**
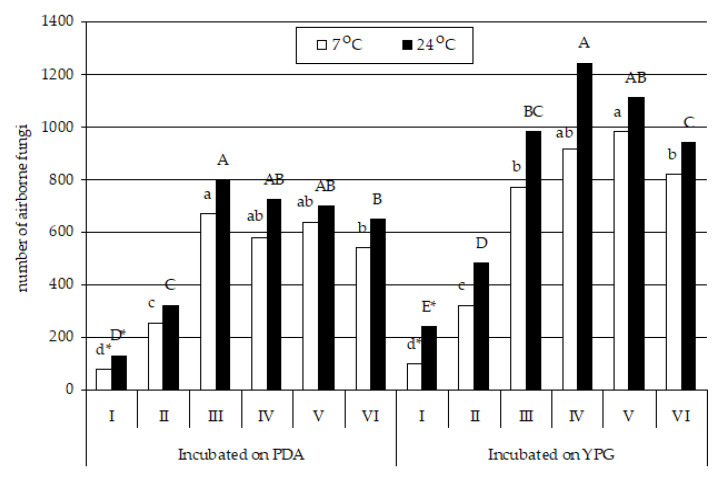
The number (CFU per 1 m^3^) of airborne fungi cultured on PDA or YPG and incubated at 7 or 24 ± 0.5 °C in the underground hibernation sites in the Nietoperek bat reserve. Study site number I was outside the underground object and locations from II to VI inside it. The air sampler was positioned 1.5 m above the level of the floor in study site numbers from I to III, and it was positioned at a distance of 0.7 to 1 m from clusters of *M. myotis* in locations from IV to VI. * For each location, the number of fungal spores followed by the same letter are not statistically different, and others are (Tukey’s HSD test, α ≤ 0.05). Letters indicate the differences between fungal species in a given location: small letters for 7 ± 0.5 °C and capital letters for 24 ± 0.5 °C; the analyses was carried out separately for PDA and YPG.

The air in the underground Nietoperek bat reserve was more contaminated by fungi than the air outside, regardless of the incubation temperature (at 7 °C, *p* _study sites I, II_ = 0.004318 for PDA and *p* _study sites I, II_ = 0.005740 for YPG; at 24 °C, *p* _study sites I, II_ = 0.001898 for PDA and *p* _study sites I, II_ = 0.000765 for YPG). However, more fungi were isolated from the air samples on the YPG medium than on the PDA medium at both sample incubation temperatures (Table A2 and Table A3 and Figure 4). Psychrophilic and psychrotolerant fungi were most abundant in study site III (*p* _study sites III, VI_ = 0.000198) on PDA and in study site V on YPG (*p* _study sites V, VI_ = 0.049506) (Table A2 and Figure 4). On the other hand, fungi isolated at the optimal growth temperature for mesophiles were most frequently isolated from study site III on PDA (*p* _study sites III, VI_ = 0.011379) and study site IV on YPG (*p* _study sites III, IV_ = 0.000443) (Table A3 and Figure 4).

*Cladosporium herbarum*, *Penicillium freii*, and *Penicillium lilacinoechinulatum* were isolated only from the outdoor air samples in all variants of the experiments. *Absidia glauca*, *Aspergillus fumigatus*, *A. tubingensis*, *Mortierella polycephala*, *Naganishia diffluens*, and *Rhodotorula mucilaginosa* were isolated only from locations IV, V, and VI under the clusters of hibernating *M. myotis*. In turn, *Penicillium chrysogenum* was the most frequently isolated species in these studies (Table A2 and Table A3). This species was dominant in study sites I and III from PDA at 7 °C, and, in study sites IV, V, and VI, it was isolated at a statistically similar level to *Penicillium crustosum*, except in study site II, where *P. concentricum* was the dominant species. *Penicillium chrysogenum* on YPG at 7 °C was the most abundant species isolated from the air, except for study site I, where this species was distributed at a statistically similar level to *P. freii* (Table A2). The species that was most abundantly cultured at the optimal growth temperature (24 °C) for mesophiles on the PDA medium in study sites no. III, IV, and VI was *P. chrysogenum*; in study site no. I, the most dominant species was *P. lilacinoechinulatum; in study site no. II, P. virdicatum* and *P. chrysogenum* were the most predominant; and, in study site no. V, the most numerously isolated species was *P. bialowiezense*. Additionally, the most abundant species found on YPG at 24 °C were *P. crustosum* in study site no. I; *P. chrysogenum*, *P. virdicatum*, and *P. brevistipitatum* in study site no. II; *P chrysogenum* and *P. crustosum* in study site no. III; *P. chrysogenum* in study site no. IV; *P. brevistipitatum* in study site no. V; and both *P. concentricum* and *P. chrysogenum* in study site no. VI (Table A3). The sites differed from each other in the diversity of the fungal species, which is illustrated by the Shannon index. The species diversity of the airborne fungi inside the cave was greater than outside, especially in the test sites where the air samples were taken from under clusters of hibernating *M. myotis* (Table A2 and Table A3).

**Figure 5:**
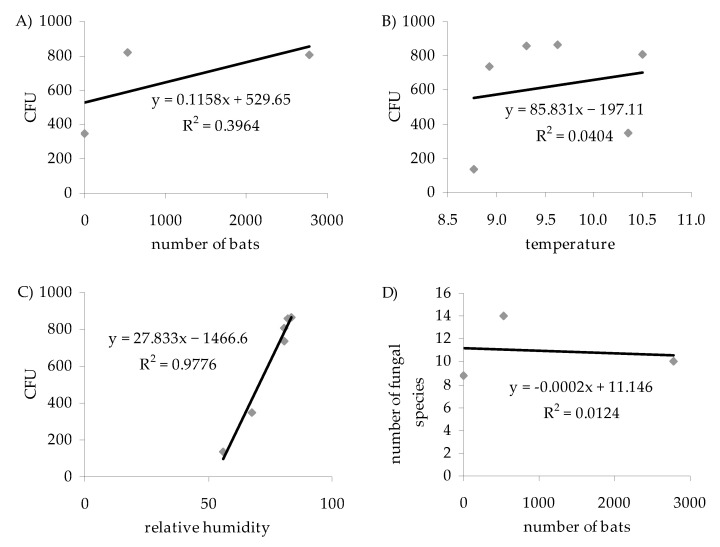
Relationships between (**A**) the number of bats and airborne fungal spores (CFU m^−3^ of air), (**B**) the number of airborne fungal spores (CFU m^−3^ of air) and temperature (°C), (**C**) the number of airborne fungal spores (CFU m^−3^ of air) and relative humidity (%), and (**D**) the number of bats and airborne fungal species in the underground hibernation sites of the Nietoperek bat reserve. Study site number I was outside the underground object and locations from II to VI inside it. An air sampler was positioned 1.5 m above the level of the floor in the study sites from I to III, and it was positioned at a distance of 0.7 to 1 m from the clusters of *M. myotis* in the locations from IV to VI.

Both the temperature and the humidity of the air, plus the number of bats, correlated positively with the concentration of airborne fungal propagules obtained on both media at both incubation temperatures (*p* <0.05; *r* = 0.20, *r* = 0.99 and *r* = 0.63, respectively) (Table 1, Table 2, Table A2, and Table A3 and Figure 5). For individual variants of the experiment, the correlation values between the temperature and the concentration of fungi in the air were (*p* < 0.05): *r* = 0.29 for 7 °C and PDA, *r* = 0.09 for 7 °C and YPG, *r* = 0.31 for 24 °C and PDA, and finally, *r* = 0.16 for 24 °C and YPG. The correlation between the air humidity and the concentration of fungal spores was (*p* < 0.05): *r* = 0.97 for 7 °C and PDA. *r* = 0.98 for 7 °C and YPG, *r* = 0.97 for 24 °C and PDA, and *r* = 0.98 for 24 °C and YPG. In turn, the correlation between the number of bats and the concentration of aeromycota was (*p* < 0.05): *r* = 0.79 for 7 °C and PDA, *r* = 0.47 for 7 °C and YPG, *r* = 0.80 for 24 °C and PDA, and *r* = 0.50 for 24 °C and YPG. Equally strong positive correlations were found between concentrations of airborne fungal propagules and the Shannon Diversity Index of the fungal species (*p* < 0.05, *r* = 0.56 for 7 °C and PDA, *r* = 0.78 for 7 °C and YPG, *r* = 0.75 for 24 °C and PDA, and *r* = 0.82 for 24 °C and YPG) (Table 1, Table 2, Table A2, and Table A3). No positive correlation was found between the number of fungal species and the number of bats (*p* < 0.05, *r* = −0.11), but the findings were inconsistent, requiring further study (Figure 5). For instance, the experiment at 24 °C (*p* < 0.05; *r* = −0.33 for PDA and *r* = −0.57 for YPG) showed no relationship, but the observations made at 7 °C show a positive correlation (*p* < 0.05; *r* = 0.45 for PDA and *r* = 0.90 for YPG) (Table 1, Table 2, Table A2, and Table A3).

## 4. Discussion

### 4.1. Physical Conditions in the Nietoperek Bat Reserve

Most underground ecosystems, both natural and anthropogenic, are heterotrophic [56], with one of the exceptions being Movile Cave “*Peștera Movile*” in Romania, which is a chemolithoautotrophic ecosystem based on sulfur. Generally, subterranean ecosystems have very specific conditions and are among the most inhospitable habitats for mycobiota, mainly due to a lack of nutrients from the absence of light and primary productivity, as well as stable low temperatures [29,56]. The Nietoperek bat reserve is a heterotrophic ecosystem, and the microclimatic conditions (temperatures: from 8.93 ± 0.07 to 10.50 ± 0.00 °C; RH: from 67.65% ± 0.59% to 82.06% ± 0.25%) prevailing during the research inside this facility were the same as in other underground facilities during the winter in Poland [15,24,57].

Currently, an important aspect of the world environmental changes is global climate change, which may also affect underground ecosystems by increasing their temperature; however, the progress of this process in subterranean ecosystems largely depends on their location and depth [58,59]. One can assume that the increase in the temperature in underground environments, as in other ecosystems, will result in changes in the bacterial and fungal communities inhabiting them and may lead to the occurrence of new diseases in mammals caused by pathogenic microbes [60,61]. Thus, the monitoring of underground ecosystems, especially those inhabited by bats, seems to be important and essential for the maintenance of biological safety in these locations and maintaining native microbiomes.

### 4.2. Fungal Species Composition and the Number of Bats

The phylum *Ascomycota* dominates in the fungal community in natural and artificial underground ecosystems worldwide, where it constitutes approximately 69% of all cultured fungi [23]. The diversity of airborne fungal species is much higher inside underground sites than outside [15,24,57], which is consistent with our results. The large variety of airborne fungi inside Nietoperek could be explained by the specific microclimatic conditions prevailing inside and outside, including the temperature and humidity of the air, as well as the presence of bats in the winter [15]. Overall, the external environment around underground sites increases the species composition of airborne fungi in these ecosystems, since most of the fungal spores enter the underground sites from the external environment, e.g., with water and air currents [35], or can be from the growth of fungi on organic resources inside the underground environment. In the winter, however, the microclimate conditions outside the underground sites and limited availability of organic matter are not conducive for the survival and development of most fungi [24], the exception being cold-adapted, psychrophilic, or psychrotolerant fungi [62]. On the other hand, the number of bats in the Nietoperek bat reserve showed a strong positive correlation with the concentration of airborne fungal propagules in the study; the higher number of bats usually corresponded to higher fungal spores in the air, which supports earlier reports by Kokurewicz et al. [15]. Therefore, it is probable that the presence of bats in the underground corridors was the main factor influencing the number and species composition of aeromycota in this study.

*Penicillium* spores were dominant in our study, regardless of the type of culture medium and the incubation temperature, which is consistent with previous research [15,24]. *Penicillium chrysogenum* was the most frequently isolated species in our current study both inside and outside the underground site. This species is cosmopolitan and often associated with bats. Ogórek et al. [36] reported that it was the most frequently isolated species from the wing membranes of female *M. myotis* during the spring emergence from the Nietoperek underground hibernation site.

Furthermore, *Absidia glauca*, *A. fumigatus*, *A. tubingensis*, *M. polycephala*, *N. diffluens*, and *R. mucilaginosa* were cultured only from the air samples taken under clusters of hibernating greater mouse-eared bats and dominated especially in the air samples taken from outside. Both *Cladosporium* and *Aspergillus* are among the most common outdoor and indoor airborne fungi [43,63]. Moreover, the fungi from these genera have been isolated from bat carcasses and from the bodies (in Italy) and wings of living bats (in USA and Poland) [21,36,64]. *Cladosporium cladosporioides* and *C. macrocarpum* were isolated from the inside air of the Nietoperek bat reserve. Both species are cosmopolitan, although the former occurs more frequently in the environment [43]. *Cladosporium cladosporioides* is often found on decaying or necrotic plant matter and bat carcasses or inhabiting leaves as a secondary invader, but it can also be cultivated from foodstuffs or soil. What is important is that it exhibits some specific and crucial survival features. It is xerophilic and capable of growing in environments with low water activity, as well as psychrophilic, therefore colonizing facilities with temperature ranges between −10 and −3 °C [64,65]. Our investigation also showed the presence of other veterinary and clinically important fungi like *R. mucilaginosa, Absidia glauca, Naganishia diffluens,* and *Mortierella polycephala*. The first was isolated from bent-winged bats in Australia [66] and found in the oral cavity and wing membranes of bats in a Brazilian cave [10]. *Naganishia diffluens* was cultured from the air, a purification tank for polluted water, and a diseased human fingernail [67]. *Mortierella polycephala* is a cosmopolitan fungus first described in 1863 by Henri Coemans as the first species of the genus [68]. This species is able to decompose chitin and was isolated from soil, dead or dying plant tissues, water, and animal excrements, including bats [69,70]. The minimal growth of this species was observed at 4 °C; therefore, it can easily inhabit the underground environment [69]. *Absidia glauca* is a species commonly found in the environment. In underground ecosystems, it was isolated from bats and their guano [15,30].

*Filobasidium magnum* and the *Mucor* genus were also isolated during our research, the former at the lower incubation temperature while the latter at the temperature optimal for mesophilic species. Interestingly, these species were found inside the underground facility and even under clusters of hibernating greater mouse-eared bats. *Filobasidium magnum* is a psychrophilic fungus; therefore, this is why it probably dominated at the lower incubation temperature [71]. In turn, the fungi of the *Mucor* genus were previously isolated from bat cadavers (*Rhinolophus affinis*) in a cave in China, and those studies indicated *M. hiemalis* as a dominant species [31], as well as from bat cadavers (*Myotis capaccini*, *Myotis* sp., *Miniopterus schreibersii*, *Pipistrellus* sp., and an undetermined bat) in Palummaro Cave in Italy [64]. Mucor is another widespread filamentous fungus that is found in soil and decaying plant matter. Three *Mucor* species (*M. circinelloides*, *M. flavus*, and *M. fragilis*) were identified inside the underground site.

### 4.3. Influence of Air Temperature and Humidity on the Number and Species Composition of Airborne Culturable Fungi

Fungal survival and diversity are strictly dependent on microclimatic conditions. Based on the previous research, the most crucial climatic factors are air temperature and humidity, as well as the quantity of organic matter [15]. The number and species composition of airborne culturable fungi also depends on other factors, such as ultraviolet radiation, pressure, and chemical atmospheric pollution [72].

The harsh and unwelcoming conditions present in underground ecosystems limit the number of microorganisms allowing the most tolerant or adaptive species to survive. The presence of bats and microorganisms inhabiting them might constitute a very small potential threat for animal and human health [15,36].

Both the temperature and the humidity of the air in the Nietoperek bat reserve correlated positively with the concentration of airborne fungal propagules in the study; the higher the temperature and humidity of the air, the more fungi were isolated. Moreover, regardless of the incubation temperature of the samples, the number of fungal species isolated from the inside of the underground facility were usually more diverse than from the outside, which is consistent with previous findings [15,73].

### 4.4. Influence of the Culture Medium and Incubation Temperature on Fungal Isolation

Incubation temperatures are also important for the in vitro microbiological analysis of biological material, since the temperature can influence the number and species composition of isolated microorganisms [16,74]. For example, most fungi grow well at room temperature (20–25 °C), but there are cold-adapted species, such as psychrophilic and psychrotrophic fungi, that prefer cooler temperatures [62]. Therefore, we used different incubation temperatures in order to obtain a wide spectrum of fungal species. More importantly, these cold-adapted fungi are often pathogenic to bats during torpor and hibernation, an example being Pd [75]. However, we did not detect this species in our research, pointing at a good health condition of bats at the Nietoperek bat reserve in a mycological context. On the other hand, another study demonstrated its presence in this underground facility [15]. The disease caused by Pd affects hibernating bats. It has caused an unprecedented bat mortality event that started in Northeastern USA and Canada [76,77]. It should also be noted that, although Pd occurs in European bats, it is not as harmful as in the USA and Canada [27], likely due to a long coevolutionary history.

The results of microbiological studies of the environment with the use of a culture-based analysis are also largely conditioned by the type of culture medium used for the analysis [78]. Various media are used for the incubation of fungi, but Sabouraud agar, YPG, and PDA are the most frequently used [15,30,79]. At the same time, it should be noted that Sabouraud agar demonstrates a comparable efficacy to PDA [80]. Therefore, we used PDA and YPG media for fungi cultured from the Nietoperek underground site in order to obtain a wide spectrum of fungal species. Our research showed that YPG was more efficient in isolating fungal species than PDA. Moreover, there were some differences in the colony count, as *Penicillium* dominated on YPG at both incubation temperatures and *Filobasidium* at 7 °C, but when PDA was used, the CFU of *Cladosporium* increased at both incubation temperatures. However, it should be noted that a culture-based analysis is more common and cheaper than molecular biological methods, but it has several disadvantages. This method cannot detect nonculturable fungi, and it may overlook fungal species that are not easily culturable. Furthermore, it might under-represent those fungal types that grow slowly because they are overtaken by faster-growing colonies [23,78]. On the other hand, it is important to perform microbial isolations of insufficiently studied environments to provide new data [20,52]. Molecular research based on metagenomics can be effective, as it offers a powerful lens for observing the microbial communities and has the potential to revolutionize our understanding of the entire living world. Although this method also has its drawbacks, including the already mentioned still high cost, it also some limitations in species identification [81].

### 4.5. Mycological Air Quality and Biological Safety Assessment for Human and Animal Health

One of the goals of our research was to evaluate the potential impact of airborne fungi on human and bat health. Overall, we confirmed that the concentration of fungi is higher inside underground ecosystems than outside during the winter [15,24,82]. Additionally, we found that, with the increasing concentration of fungal spores, the number of isolated fungal species grew. Although there are no official mycological air quality standards relating specifically to underground sites, such requirements exist with respect to the indoor air of buildings. For example, based on the Polish norm PN89/Z-04111/03, air containing up to 3000 CFU of fungal spores in 1 m^3^ is considered to be “uncontaminated” [83]. However, the World Health Organization (WHO) stated that the concentration of 1500 CFU airborne fungi in 1 m^3^ of air is acceptable but only in the case of a mixture of species [84]. Thus, the concentration of airborne fungi in the Nietoperek bat reserve was within these ranges, both according to the Polish norm and the WHO directive, and does not pose a threat for human health. At 7 ± 0.5 °C, the highest concentration of airborne fungi inside the underground facility was 673 CFU · m^−3^ on PDA medium and 981 CFU per 1 m^3^ on YPG medium, while, at 24 ± 0.5 °C, the concentrations were higher, especially on the YPG medium, which was 125 CFU per 1 m^3^, and 798 CFU per 1 m^3^ on PDA. Moreover, the concentration of fungal spores obtained in our research was at similar levels as in other underground sites in Poland inhabited or not inhabited by bats studied during the winter using an air sampler and PDA medium [15,24,57].

We did not find the most dangerous pathogen of hibernating bats (Pd, the etiological agent of WNS), which was previously detected in the Nietoperek reserve [15]. The most abundant species occurring in our research may pose a potential biological threat. Fungi belonging to the *Penicillium* genus can be extremely dangerous to humans and other mammals due to their great potential for mycotoxin secretion and the production of conidial spores [20,38]. Therefore, *Penicillium* and *Aspergillus* fungi are among the most important biological factors contributing to sick building syndrome, where people develop symptoms or chronic infections from being in the building [85]. For example, among isolated *Penicillium* species, *P. viriditacum* synthesize at least four different toxins (ochratoxin A, rubrosulphin, viopurpurin, and viomellein), of which ochratoxin A is a carcinogen and teratogen, as well as immuno- and nephrotoxic agents that are especially dangerous to humans and animals [86,87]. Most isolates of *P. expansum* produce a highly toxic mixture of patulin, citrinin, chaetoglobosins, and communesins, as well as the less toxic roquefortine C [88]. In turn, *P. crustosum* produces the neurotoxic penitrems, including penitrems A–G; it can also produce thomitrems A and E and roquefortine C [89]. The most toxic metabolite secreted by *P. brevicompactum* is the mutagenic compound botryodiploidin [90]. On the other hand, *P. chrysogenum* is a less common fungal pathogen, and its toxicity is limited. However, it may be hazardous to people with a weakened immune system, causing pneumonia [91]. It has also been reported in the first case of invasive pulmonary mycosis in a lung transplant recipient [92]. *Aspergillus* species in general can cause numerous, often serious, human diseases [93]. For example, *A. fumigatus*, which was isolated in our study under the clusters of hibernating bats, is an infectious agent associated with severe and often fatal infections, especially in immunocompromised patients, and it is classified in risk group 2 regarding pathogenic fungi [94,95]. This species primarily attacks the lungs; however, it can also cause allergic bronchopulmonary aspergillosis, chronic lung infections, or allergies [94]. Therefore, *A. fumigatusis* is probably the most dangerous type of *Aspergillus* [95]. *Aspergillus tubingensis*, also associated with *M. myotis* in our research, is mostly a cause of fungal keratitis [96]. Other fungi that were isolated only under the clusters of hibernating bats are also of clinical importance. Namely, *R. mucilaginosa* is an emerging human pathogen causing bloodstream and central nervous system infections [97], and *M. polycephala* may cause pulmonary mycosis in cattle [98]. In turn, *N. diffluens* might cause skin infections [99].

Another noticeable group of fungi found in our research, especially outside, was *Cladosporium*. This genus is commonly classified as an indoor and outdoor mold. These species are characterized by a cosmopolitan distribution and can inhabit various debris or be cultured from soils, textiles, paint, plants, and food [43,63]. Their conidia are compact in size and spread easily over long distances. *Cladosporium* spores are one of the most allergenic biological particles in the air, which can cause allergic rhinitis, asthma, or allergic alveolitis. However, a minimum of 2800 spores of this fungi in one cubic meter of air are required for the emergence of respiratory allergies in humans [100]. The concentration of *Cladosporium* spores in our study were at much lower levels—up to 126 CFU per 1 m^3^ of air. *Cladosporium cladosporioides*, which was isolated inside the Nietoperek bat reserve, is rarely a cause of disease in humans; however, some studies reported rare acute infections [99,101]. This species can also cause asthmatic reactions to allergens, as well as beta-glucans present on the spore surface, and it may also increase respiratory inflammation [102]. Nearly any fungus can cause an infection in a severely immunocompromised patient, caused by the treatment for cancer or other conditions causing immunodeficiency, AIDS, or a transplant recipient. Most of the fungi we isolated are not a threat to the average tourist, and the low numbers of spores further reduce the health risk.

## 5. Conclusions

Our study contributes to gaining new knowledge about the aeromycota present in the close vicinity of hibernating *Myotis myotis* in an underground site at the Nietoperek bat preserve in Poland. Overall, we isolated 32 fungal species, including cosmopolitan species such as *Penicillium chrysogenum*. Most importantly, we detected species closely associated with bats, which were isolated only under clusters of hibernating *M. myotis*: *Absidia glauca*, *Aspergillus fumigatus*, *Aspergillus tubingensis*, *Mortierella polycephala*, *Naganishia diffluens*, and *Rhodotorula mucilaginosa*. We did not find the most dangerous pathogen of hibernating bats (Pd), although it has been detected at this site in the past. There are many reasons why Pd was not detected, but the two most likely are the accuracy of the test method or lack of Pd during the test. Pd is a slow-growing fungal species [25], and it is often overgrown by other fast-growing species in culture-based analyses [74]. On the other hand, we did not observe the characteristic symptoms of Pd on bats [76] during the study. Hibernating *M. myotis* contributed to an increase in the concentration of aeromycota in the underground site and is as a vector/reservoir of microscopic fungi, including those with that are potential threats to mammal populations. Therefore, the summer period seems to be the best time to visit underground ecosystems where bats hibernate, because during this period, they are not found in underground sites but in their summer breeding shelters. We also showed that YPG is a better medium for aeromycological research than PDA, because it gave higher fungal isolations. The number of fungal species correlated positively with the concentration of airborne fungal propagules but did not positively correlate with the number of bats. However, we confirmed the current knowledge that the temperature and the humidity of the air, as well as the number of bats, correlated positively with the concentration of airborne fungal propagules obtained. The air in the underground site was more contaminated by fungi than the air outside during the winter. Currently, the risk to tourists visiting the Nietoperek bat reserve is very low, and no action needs to be taken. Increasing tourism will lead to a better understanding of bats and their role in the ecosystem and the economy [5] and the role of hibernation in their conservation [8]. To ensure future safety, aeromycological monitoring should continue.

## Figures and Tables

**Table 1:** Study sites and average values of microclimatic conditions in parts of the Nietoperek bat reserve underground investigated for aeromycology: ^1^ See Figure 1 for locations. ^2^ SD (standard deviation). ^3^ For each location, microclimatic conditions followed by the same letter are not statistically different at the α ≤ 0.05 level, according to Tukey’s HSD test; others are. Letters indicate the effect of location on particular conditions in the Nietoperek bat reserve; they refer to means along the columns.

Study Site Number	Locations ^1^	Section No. ^1^	Dimensions of Underground Corridors				Temperature (°C) ± SD ^2^ (*n* = 9)		Relative Humidity (%) ± SD (*n* = 9)	
			Length (m)	Height (m)	Width (m)	Volume (m^3^)				
I	Entrance to object	near 7.10.2	―	―	―	―	8.77 ± 0.07	f ^3^	56.20 ± 0.24	e
II	from security gate to Bhf Heinrich	7.8	669.0	2.0	2.2	2943.6	10.35 ± 0.05	b	67.65 ± 0.59	d
III	from Bhf Gustav to Bhf Friedrich	7.5	1075.0	3.2	3.7	12,728.0	10.50 ± 0.00	a	80.68 ± 0.07	c
IV	from Bhf Friedrich of Section 7.2	beginning of 7.2	806.0	2.0	2.2	3546.4	9.63 ± 0.10	c	83.72 ± 0.61	a
V		center of 7.2					9.31 ± 0.05	d	82.06 ± 0.25	b
VI		end of 7.2					8.93 ± 0.07	e	80.63 ± 0.04	c

**Table 2:** The number and species composition of the bats found in parts of the Nietoperek bat reserve underground investigated for aeromycology.

Bat Species	Number of Bats		
	No. 7.2 (Study Site No. IV, V, and VI)	No. 7.5 (Study Site No. III)	No. 7.8 (Study Site No. II)
*Myotis myotis*	497	2052	3
*Myotis daubentonii*	20	411	1
*Myotis nattereri*	15	287	0
*Barbastella barbastellus*	0	2	0
*Plecotus auritus*	4	21	0
*Myotis dasycneme*	0	2	0
Other species	1	0	0
In total	537	2775	4
In total per 1 m^3^ of the corridor	0.151	0.218	0.001
% of *M. myotis* to the total bat number	92.6	73.9	75.0

## Data Availability

Not applicable.

## Appendix A

**Table A1:** BLAST analysis of rDNA ITS of culturable aeromycota isolated during research in the underground hibernation sites of the Nietoperek bat reserve and/or present close to hibernating *Myotis myotis*. All E values were zero.

Fungi	Isolate	GenBank Accession No.	Identity with Sequence from GenBank			
			Query Cover, %	Identity, %	Accession	Isolate
*Absidia glauca*	UWR_114	MK690542.1	99%	94.71%	KY465754.1	UWR_100
*Aspergillus fumigatus*	UWR_115	MK690543.1	100%	99.72%	MN258567.1	R29
*Aspergillus tubingensis*	UWR_116	MK690544.1	100%	100.00%	KU243047.1	HRb
*Botrytis cinerea*	UWR_117	MK690545.1	100%	100.00%	MG744435.1	MGGM002
*Cladosporium cladosporioides*	UWR_118	MK690546.1	99%	99.80%	JN986781.1	DHMJ29
*Cladosporium herbarum*	UWR_119	MK690547.1	100%	100.00%	MN486496.1	YMZZ2
*Cladosporium macrocarpum*	UWR_120	MK690548.1	100%	99.60%	KX815295.1	JS4-25
*Debaryomyces hansenii*	UWR_121	MK690549.1	100%	99.83%	MK268125.1	R97192
*Neoascochyta exitialis*	UWR_122	MK690550.1	100%	99.60%	EU167564.1	CBS 446.82
*Filobasidium magnum*	UWR_123	MK690551.1	100%	100.00%	MK226208.1	D6
*Mortierella polycephala*	UWR_124	MK690552.1	99%	99.16%	MH860490.1	CBS 327.72
*Mucor circinelloides*	UWR_125	MK690553.1	100%	100.00%	MK928427.1	KKP 3008
*Mucor flavus*	UWR_126	MK690554.1	94%	92.93%	NR_103633.1	CBS 234.35
*Mucor fragilis*	UWR_127	MK690555.1	100%	99.66%	MK910073.1	BC3
*Naganishia diffluens*	UWR_128	MK690556.1	100%	100.00%	KY104328.1	CBS:160
*Penicillium bialowiezense*	UWR_129	MK690557.1	100%	100.00%	MH854996.1	CBS 227.28
*Penicillium brevicompactum*	UWR_130	MK690558.1	100%	100.00%	MH865309.1	CBS 129415
*Penicillium brevistipitatum*	UWR_131	MK690559.1	99%	99.62%	MG490883.1	KAS7539
*Penicillium cavernicola*	UWR_132	MK690560.1	100%	100.00%	MN413150.1	CBS 109557
*Penicillium chrysogenum*	UWR_133	MK690561.1	99%	100.00%	KM357336.1	no data
*Penicillium commune*	UWR_134	MK690562.1	99%	100.00%	MK660354.1	QP3
*Penicillium camemberti*	UWR_135	MK690563.1	100%	99.62%	KY218668.1	IF2SW-F1
*Penicillium concentricum*	UWR_136	MK690564.1	100%	100.00%	DQ339561.1	NRRL 2034
*Penicillium crustosum*	UWR_137	MK690565.1	100%	91.00%	KF938379.1	04SK002
*Penicillium echinulatum*	UWR_138	MK690566.1	100%	100.00%	KT876710.1	A4-2
*Penicillium expansum*	UWR_139	MK690567.1	100%	100.00%	MK201595.1	SE1
*Penicillium freii*	UWR_140	MK690568.1	99%	99.44%	JN942696.1	CBS 794.95
*Penicillium lilacinoechinulatum*	UWR_141	MK690569.1	100%	99.63%	KC773837.1	CBS 454.93
*Penicillium robsamsonii*	UWR_142	MK690570.1	100%	99.63%	NR_144866.1	CBS 140573
*Penicillium solitum*	UWR_143	MK690571.1	100%	100.00%	MK660356.1	QPA4
*Penicillium viridicatum*	UWR_144	MK690572.1	100%	100.00%	MH856411.1	CBS 390.48
*Rhodotorula mucilaginosa*	UWR_145	MK690573.1	100%	100.00%	MK263185.1	IMUFRJ 52392

**Table A2:** The number (CFU m^−3^ of air, *n* = 6) and species composition of airborne fungi cultured at 7 ± 0.5 °C during research in the underground hibernation sites in the Nietoperek bat reserve (study site numbers from I to III) and/or present close (study site numbers from IV to VI) to hibernating *Myotis myotis*. Study site number I was outside the underground object and locations from II to VI inside it. An air sampler was positioned 1.5 m above the level of the floor in study site numbers from I to III, and it was positioned at a distance of 0.7–1 m from clusters of *M. myotis* in locations from IV to VI: ^1^ ”―” means not detected. ^2^Statistical analysis was performed only for fungal species that were detected in a given study site. For each location, the number of fungal spores followed by the same letter are not statistically different, and others are (Tukey’s HSD test, α ≤ 0.05). Letters indicate the differences between fungal species in a given location; they refer to means in the columns. All analyses were carried out separately for PDA and YPG.

Fungi	Incubated on PDA												Incubated on YPG											
	I		II		III		IV		V		VI		I		II		III		IV		V		VI	
*Cladosporium macrocarpum*	23	ab ^2^	5	c	109	ab	126	ab	38	b	46	b	4	b ^2^	―		34	cde	80	bc	40	d	55	b
*Neoascochyta exitialis*	― ^1^		―		17	d	21	d	9	b	―		― ^1^		―		2	e	―		―		―	
*Filobasidium magnum*	14	bc	―		76	bc	39	cd	13	b	34	b	10	b	―		133	b	91	bc	253	b	301	a
*Mucor circinelloides*	―		―		―		―		―		―		―		―		21	de	―		―		―	
*Mucor fragilis*	―		―		―		―		―		―		―		―		4	e	25	c	14	d		
*Penicillium chrysogenum*	31	a	69	b	228	a	166	a	201	a	213	a	35	a	221	a	307	a	506	a	442	a	376	a
*Penicillium concentricum*	―		154	a	96	bc	―		52	b	―		―		87	b	76	bcd	―		29	d	―	
*Penicillium crustosum*	―		26	bc	87	bc	149	a	306	a	248	a	―		6	c	92	bc	46	bc	165	c	90	b
*Penicillium freii*	9	c	―		―		―		―		―		53	a	―		―		―		―		―	
*Penicillium solitum*	―		―		60	bc	78	bc	11	b	―		―		8	c	105	b	167	b	38	d	―	
*Rhodotorula mucilaginosa*	―		―		―		2	cd	6	b	―		―		―		―		―		―		―	
Shannon Index	0.5594		0.4204		0.7637		0.7074		0.5833		0.4812		0.4612		0.3378		0.5290		0.5773		0.6305		0.4989	

**Table A3:** The number (CFU m^−3^ of air, *n* = 6) and species composition of airborne fungi cultured at 24 ± 0.5 °C during research in the underground hibernation Nietoperek bat reserve (study site numbers from I to III) and/or present close (study site numbers from IV to VI) to hibernating *Myotis myotis*. Study site number I was outside the underground object and locations from II to VI inside it. An air sampler was positioned 1.5 m above the level of the floor in study site numbers from I to III, and it was positioned at a distance of 0.7–1 m from clusters of *M. myotis* in locations from IV to VI: ^1^ ”―” means not detected. ^2^ Statistical analysis was performed only for fungal species that were detected in a given study site. For each location, the number of fungal spores followed by the same letter are not statistically different, and others are (Tukey’s HSD test, α ≤ 0.05). Letters indicate the differences between fungal species in a given location; they refer to means in the columns. All analyses were carried out separately for PDA and YPG.

Fungi	Incubated on PDA												Incubated on YPG											
	I		II		III		IV		V		VI		I		II		III		IV		V		VI	
*Absidia glauca*	― ^1^		―		―		7	ef	―		11	d	― ^1^		―		―		15	e	24	cd	59	bcd
*Aspergillus fumigatus*	―		―		―		―		―		―		―		―		―		36	e	9	d	44	bcdef
*Aspergillus tubingensis*	―		―		―		6	ef	7	f	4	d	―		―		―		2	e	7	d	3	ef
*Botrytis cinerea*	7	c ^2^	6	d	5	e	―		11	f	22	cd	21	b ^2^	3	b	―		―		18	cd	30	cdef
*Cladosporium cladosporioides*	32	ab	24	bcd	40	de	11	ef	27	ef	38	bcd	12	b	5	b	44	bcd	13	e	10	d	―	
*Cladosporium herbarum*	23	bc	―		―		―		―		―		5	b	―		―		―		―		―	
*Cladosporium macrocarpum*	―		―		63	cde	9	ef	―		15	d	―		11	b	36	bcd	55	de	―		43	bcdef
*Debaryomyces hansenii*	―		1	d	―		2	f	―		3	d	―		―		―		3	e	―		1	f
*Mortierella polycephala*	―		―		―		15	ef	40	def	―		―		―		―		―		―		―	
*Mucor circinelloides*	―		9	cd	34	de	18	def	―		59	bc	―		26	b	―		35	e	51	cde	66	bc
*Mucor flavus*	―		―		―		―		―		―		―		―		78	bc	―		―		18	def
*Mucor fragilis*	―		―		―		―		―		―		―		―		―		―		25	de	21	cdef
*Naganishia diffluens*	―		―		―		―		73	cde	12	d	―		―		―		―		―		17	def
*Penicillium bialowiezense*	―		40	ab	106	bc	110	b	164	a	62	bc	―		22	b	80	bc	―		114	bc	―	
*Penicillium brevicompactum*	―		47	ab	137	b	59	cd	87	cd	78	b	―		―		―		―		―		―	
*Penicillium brevistipitatum*	―		―		42	de	―		―		―		―		80	a	55	bcd	138	bcd	245	a	41	bcdef
*Penicillium cavernicola*	―		―		―		―		―		―		―		12	b	―		87	cde	43	de	56	bcd
*Penicillium chrysogenum*	12	c	65	a	198	a	229	a	110	bc	184	a	2	b	121	a	219	a	284	a	165	b	145	a
*Penicillium commune*	―		―		―		―		―		―		―		3	b	12	d	21	e	72	cde	50	bcde
*Penicillium camemberti*	―		―		―		―		―		―		―		8	b	―		―		―		7	ef
*Penicillium concentricum*	―		35	bc	―		37	def	―		30	cd	8	b	4	b	―		179	bc	―		169	a
*Penicillium crustosum*	―		10	cd	70	cd	83	bc	150	ab	61	bc	171	a	31	b	179	a	85	de	48	cde	82	b
*Penicillium echinulatum*	―		21	bcd	―		60	cd	―		43	bcd	―		33	b	63	bcd	44	e	78	cd	―	
*Penicillium expansum*	―		―		―		―		―		―		―		―		21	cd	26	e	27	de	―	
*Penicillium freii*	6	c	―		―		―		―		―		4	b	―		―		―		―		―	
*Penicillium lilacinoechinulatum*	51	a	―		―		―		―		―		19	b	―		―		―		―		―	
*Penicillium robsamsonii*	―		―		―		―		―		―		―		9	b	47	bcd	―		21	cd	25	cdef
*Penicillium solitum*	―		―		47	cde	29	def	―		―		―		2	b	53	bcd	41	e	―		―	
*Penicillium viridicatum*	―		63	a	56	cde	48	cde	30	ef	26	cd	―		115	a	96	b	181	b	156	b	64	bcd
Shannon Index	0.6661		0.9113		0.9359		0.9393		0.8654		0.9974		0.4806		0.9260		1.0047		1.0232		1.0440		1.1283	

## References

[1] Jones G., Jacobs D.S., Kunz T.H., Willig M.R., Racey P.A. (2009). Carpe Noctem: The importance of bats as bioindicators. Endanger. Species Res..

[2] Mildenstein T., Tanshi I., Racey P.A. (2016). Exploitation of bats for bushmeat and medicine. Bats in the Anthropocene: Conservation of Bats in a Changing World.

[3] Shetty S., Sreepada K.S., Bhat R. (2013). Effect of bat guano on the growth of *Vigna radiata* L. Int. J. Sci. Res. Publ..

[4] Boyles J.G., Cryan P.M., McCracken G.F., Kunz T.H. (2011). Economic importance of bats in agriculture. Science.

[5] Vilas R.A. (2016). Ecological and economical impact of bats on ecosystem. Int. J. Life Sci..

[6] Berková H., Pokorný M., Zukal J. (2014). Selection of buildings as maternity roosts by greater mouse-eared bats (*Myotis myotis*). J. Mammal..

[7] Bagstad K.J., Wiederholt R. (2013). Tourism Values for Mexican Free-Tailed Bat Viewing. Hum. Dimens. Wildl..

[8] Pennisi L.A., Holland S.M., Stein T.V. (2004). Achieving bat conservation through tourism. J. Ecotourism.

[9] Lyon G.M., Bravo A.V., Espino A., Lindsley M.D., Gutierrez R.E., Rodriguez I., Corella A., Carrillo F., McNeil M.M., Warnock D.W. (2004). Histoplasmosis associated with exploring a bat-inhabited cave in Costa Rica, 1998–1999. Am. J. Trop Med. Hyg..

[10] Cunha A.O.B., Bezerra J.D.P., Oliveira T.G.L., Barbier E., Bernard E., Machado A.R., Souza-Motta C.M. (2020). Living in the dark: Bat caves as hotspots of fungal diversity. PLoS ONE.

[11] Gerbáčová K., Maliničová L., Kisková J., Maslišová V., Uhrin M., Pristaš P. (2020). The faecal microbiome of building-dwelling insectivorous bats (*Myotis myotis* and *Rhinolophus hipposideros*) also contains antibiotic-resistant bacterial representatives. Curr. Microbiol..

[12] Mollentze N., Streicker D.G. (2020). Viral zoonotic risk is homogenous among taxonomic orders of mammalian and avian reservoir hosts. Proc. Natl. Acad. Sci. USA.

[13] Mallapaty S. (2020). Meet the scientists investigating the origins of the COVID pandemic. Nature.

[14] Kretzschmar F., Heinz B. (1995). Social behaviour of a large population of *Pipistrellus pipistrellus* (Schreber, 1774) (Chiroptera: Vespertilionidae) and some other bat species in the mining-system of a limestone quarry near Heidelberg (South West Germany). Myotis Int. J. Bat Res..

[15] Kokurewicz T., Ogórek R., Pusz W., Matkowski K. (2016). Bats increase the number of cultivable airborne fungi in the “Nietoperek” bat reserve in Western Poland. Microb. Ecol..

[16] Ogórek R., Guz-Regner K., Kokurewicz T., Baraniok E., Kozak B. (2018). Airborne bacteria cultivated from underground hibernation sites in the Nietoperek bat reserve (Poland). J. Caves Karst Stud..

[17] Watson C. (2020). Bats are a key source of human viruses—But they’re not special. Nature.

[18] Kokurewicz T. (2004). Sex and age-related habitat selection and mass dynamics of Daubenton’s bats *Myotis daubentonii* (Kuhl, 1817) hibernating in natural conditions. Acta Chiropterol..

[19] Ogórek R., Lejman A., Matkowski K. (2013). The fungi isolated from the Niedźwiedzia Cave in Kletno (Lower Silesia, Poland). Int. J. Speleol..

[20] Jurado V., Laiz L., Rodriguez-Nava V., Boiron P., Hermosin H., Sanchez-Moral S., Saiz-Jimenez C. (2010). Pathogenic and opportunistic microorganisms in caves. Int. J. Speleol..

[21] Johnson L.J., Miller A.N., McCleery R.A., McClanahan R., Kath J.A., Lueschow S., Porras-Alfaro A. (2013). Psychrophilic and psychrotolerant fungi on bats and the presence of *Geomyces* spp. on bat wings prior to the arrival of white nose syndrome. Appl. Environ. Microbiol..

[22] Khizhnyak S.V., Tausheva I.V., Berezikova A.A., Nesterenko E.V., Rogozin D.Y. (2003). Psychrophilic and Psychrotolerant Heterotrophic Microorganisms of Middle Siberian Karst Cavities. Russ. J. Ecol..

[23] Vanderwolf K.J., Malloch D., McAlpine D.F., Forbes G.J. (2013). A world review of fungi, yeasts, and slime molds in caves. Int. J. Speleol..

[24] Ogórek R., Pusz W., Zagożdżon P.P., Kozak B., Bujak H. (2017). Abundance and diversity of psychrotolerant cultivable mycobiota in winter of a former aluminous shale mine. Geomicrobiol. J..

[25] Verant M.L., Boyles J.G., Waldrep W., Wibbelt G., Blehert D.S. (2012). Temperature-dependent growth of *Geomyces destructans*, the fungus that causes bat white-nose syndrome. PLoS ONE.

[26] Lorch J.M., Muller L.K., Russell R.E., O’Connor M., Lindner D.L., Blehert D.S. (2013). Distribution and environmental persistence of the causative agent of White-Nose Syndrome, *Geomyces destructans*, in bat hibernacula of the Eastern United States. Environ. Microbiol..

[27] Zukal J., Bandouchova H., Brichta J., Cmokova A., Jaron K.S., Kolarik M., Kovacova V., Kubátová A., Nováková A., Orlov O. (2016). White-nose syndrome without borders: *Pseudogymnoascus destructans* infection tolerated in Europe and Palearctic Asia but not in North America. Sci. Rep..

[28] Veselská T.K., Homutová K., García F.P., Kubátová A., Martínková N., Pikula J., Kolařík M. (2020). Comparative eco-physiology revealed extensive enzymatic curtailment, lipases production and strong conidial resilience of the bat pathogenic fungus *Pseudogymnoascus destructans*. Sci. Rep..

[29] Poulson T.L., Lavoie K.H., Wilkens H., Culver D.C., Humphreys W.F. (2000). The trophic basis of subsurface ecosystems. Ecosystems of the World: Subterranean Ecosystems.

[30] Ogórek R., Dyląg M., Kozak B., Višňovská Z., Tancinová D., Lejman A. (2016). Fungi isolated and quantified from bat guano and air in Harmanecka’ and Driny Caves (Slovakia). J. Caves Karst Stud..

[31] Karunarathna S.C., Dong Y., Karasaki S., Tibpromma S., Hyde K.D., Lumyong S., Xu J.C., Sheng J., Mortimer P.E. (2020). Discovery of novel fungal species and pathogens on bat carcasses in a cave in Yunnan Province, China. Emerg. Microbes Infect..

[32] Mandal J., Brandl H. (2011). Bioaerosols in indoor environments—A review with special reference to residential and occupational locations. Open Environ. Biol. Monit. J..

[33] Bastian F., Alabouvette C., Saiz-Jimenez C. (2009). The impact of arthropods on fungal community structure in Lascaux Cave. J. Appl. Microbiol..

[34] Griffin D.W., Gray M.A., Lyles M.B., Northup D.E. (2014). The transport of nonindigenous microorganisms into caves by human visitation: A case study at Carlsbad Caverns National Park. Geomicrobiol. J..

[35] Ogórek R., Lejman A., Matkowski K. (2014). Influence of the external environment on airborne fungi isolated from a cave. Pol. J. Environ. Stud..

[36] Ogórek R., Kurczaba K., Cal M., Apoznański G., Kokurewicz T. (2020). A culture-based ID of micromycetes on the wing membranes of Greater mouse-eared bats (*Myotis myotis*) from the “Nietoperek” site (Poland). Animals.

[37] Vanderwolf K.J., Campbell L.J., Goldberg T.L., Blehert D.S., Lorch J.M. (2020). Skin fungal assemblages of bats vary based on susceptibility to white-nose syndrome. ISME J..

[38] Grajewski J., Twarożek M. (2004). The healthy aspects of the influence of moulds and mycotoxins. Alergia.

[39] Frick W.F., Kingston T., Flanders J. (2020). A review of the major threats and challenges to global bat conservation. Ann. N. Y. Acad. Sci..

[40] European Commission (1992). Directive 92/43/EEC—The Conservation of Natural Habitats and of Wild Fauna and Flora in The Habitats Directive. http://ec.europa.eu/environment/nature/legislation/habitatsdirective/index_en.htm.

[41] De Bruyn L., Gyselings R., Kirkpatrick L., Rachwald A., Apoznański G., Kokurewicz T. (2021). Temperature driven hibernation site use in the Western barbastelle *Barbastella barbastellus* (Schreber, 1774). Sci. Rep..

[42] Kokurewicz T., Apoznański G., Gyselings R.L., Kirkpatrick L., De Bruyn L., Haddow J., Glover A., Schofield H., Schmidt C., Bongers F. (2019). 45 years of bat study and conservation in Nietoperek bat reserve (Western Poland). Nyctalus.

[43] Bensch K., Braun U., Groenewald J.Z., Crous P.W. (2012). The genus *Cladosporium*. Stud. Mycol..

[44] Frisvad J.C., Samson R.A. (2004). Polyphasic taxonomy of *Penicillium* subgenus *Penicillium*. A guide to identification of food and air-borne terverticillate *Penicillia* and their mycotoxins. Stud. Mycol..

[45] Chilvers M.I., du Toit L.J. (2006). Detection and identification of *Botrytis* species associated with neck rot, scape blight, and umbel blight of onion. Plant. Health Prog..

[46] Samson R.A., Visagie C.M., Houbraken J., Hong S.B., Hubka V., Klaassen C.H.W., Perrone G., Seifert K.A., Susca A., Tanney J.B. (2014). Phylogeny, identification and nomenclature of the genus *Aspergillus*. Stud. Mycol..

[47] Chen Q., Jiang J.R., Zhang G.Z., Cai L., Crous P.W. (2015). Resolving the *Phoma enigma*. Stud. Mycol..

[48] Visagie C.M., Hirooka Y., Tanney J.B., Whitfield E., Mwange K., Meijer M., Amend A.S., Seifert K.A., Samson R.A. (2014). *Aspergillus*, *Penicillium* and *Talaromyces* isolated from house dust samples collected around the world. Stud. Mycol..

[49] Doyle J.J., Doyle J.L. (1987). A rapid DNA isolation procedure for small quantities of fresh leaf tissue. Phytochem. Bull..

[50] Ogórek R., Piecuch A., Višňovská Z., Cal M., Niedźwiecka K. (2019). First report on the occurence of dermatophytes of *Microsporum cookei* clade and close affinities to *Paraphyton cookei* in the Harmanecká Cave (Veľká Fatra Mts., Slovakia). Diversity.

[51] White T.J., Bruns T., Lee S., Taylor J.W., Innis M.A., Gelfand D.H., Sninsky J.J., White T.J. (1990). Amplification and direct sequencing of fungal ribosomal RNAgenes for phylogenetics. PCR Protocols: A Guide to Methods and Applications.

[52] Ogórek R., Dyląg M., Kozak B. (2016). Dark stains on rock surfaces in Driny Cave (Little Carpathian Mountains, Slovakia). Extremophiles.

[53] Dietz C., von Helversen O. (2004). Illustrated Identification Key to the Bats of Europe.

[54] Bliss C.I. (1934). The method of probits. Science.

[55] Spellerberg I.F., Fedor P. (2003). A tribute to Claude Shannon (1916–2001) and a plea for more rigorous use of species richness, species diversity and the ‘Shannon–Wiener’ Index. Glob. Ecol. Biogeogr..

[56] Kumaresan D., Wischer D., Stephenson J., Hillebrand-Voiculescu A., Murrell J.C. (2014). Microbiology of Movile Cave—Chemolithoautotrophic ecosystem. Geomicrobiol. J..

[57] Pusz W., Baturo-Cieśniewska A., Zagożdżon P.P., Ogórek R. (2017). Mycobiota of the disused ore mine of Marcinków in Śnieżnik Masiff (western Poland). J. Mt. Sci..

[58] Domínguez-Villar D., Lojen S., Krklec K. (2015). Is global warming affecting cave temperatures? Experimental and model data from a paradigmatic case study. Clim. Dyn..

[59] Mammola S., Piano E., Cardoso P., Vernon P., Domínguez-Villar D., Culver D.C., Pipan T., Isaia M. (2019). Climate change going deep: The effects of global climatic alterations on cave ecosystems. Anthr. Rev..

[60] Garcia-Solache M.A., Casadevall A. (2010). Global warming will bring new fungal diseases for mammals. mBio.

[61] Nadkarni N.M., Solano R. (2002). Potential effects of climate change on canopy communities in a tropical cloud forest: An experimental approach. Oecologia.

[62] Wang M., Tian J., Xiang M., Liu X. (2017). Living strategy of cold-adapted fungi with the reference to several representative species. Mycology.

[63] Piecuch A., Ogórek R. (2020). Quantitative and qualitative assessment of mycological air pollution in a dormitory bathroom with high humidity and fungal stains on the ceiling. Case Study. Pol. J. Environ. Stud..

[64] Voyron S., Lazzari A., Riccucci M., Calvini M., Varese G.C. (2011). First mycological investigations on Italian bats. Hystrix.

[65] Deshmukh S.K., Rai M.K. (2005). Biodiversity of Fungi: Their Role in Human Life.

[66] Holz P.H., Lumsden L.F., Marenda M.S., Browning G.F., Hufschmid J. (2018). Two subspecies of bent-winged bats (*Miniopterus orianae bassanii* and *oceanensis*) in southern Australia have diverse fungal skin flora but not *Pseudogymnoascus destructans*. PLoS ONE.

[67] Fotedar R., Kolecka A., Boekhout T., Fell J.W., Anand A., Al Malaki A., Zeyara A., Al Marri M. (2018). *Naganishia qatarensis* sp. nov., a novel basidiomycetous yeast species from a hypersaline marine environment in Qatar. Int. J. Syst. Evol. Microbiol..

[68] Coemans E. (1863). Quelques hyphomycetes nouveaux. 1. Mortierella polycephala et Martensella pectinata. Bull. Acad. R. Sci. Belg..

[69] Dauphin J. (1908). Contribution à l’étude de Mortiérellées. Ann. Sci. Nat. Bor..

[70] Hyde K.D., Hongsanan S., Jeewon R., Bhat D.J., Mckenzie E.H.C., Gareth Jones E.B., Phookamsak R., Ariyawansa H., Boonmee S., Zhao Q. (2016). Fungal diversity notes 367–490: Taxonomic and phylogenetic contributions to fungal taxa. Fungal Divers..

[71] Aboutalebian S., Mahmoudi S., Okhovat A., Khodavaisy S., Mirhendi H. (2020). Otomycosis due to the rare fungi *Talaromyces purpurogenus*, *Naganishia albida* and *Filobasidium magnum*. Mycopathologia.

[72] Niazi S., Hassanvand M.S., Mahvi A.H., Nabizadeh R., Alimohammadi M., Nabavi S., Faridi S., Dehghani A., Hoseini M., Moradi-Joo M. (2015). Assessment of bioaerosol contamination (bacteria and fungi) in the largest urban wastewater treatment plant in the Middle East. Environ. Sci. Pollut. Res..

[73] Ogórek R. (2018). Speleomycology of air in Demänovská Cave of Liberty (Slovakia) and new airborne species for fungal sites. J. Cave Karst Stud..

[74] Marshall V., Poulson-Cook S., Moldenhauer J. (1998). Comparative mold and yeast recovery analysis (the effect of differing incubation temperature ranges and growth media). PDA J. Pharm. Sci. Technol..

[75] Minnis A.M., Lindner D.L. (2013). Phylogenetic evaluation of *Geomyces* and allies reveals no close relatives of *Pseudogymnoascus destructans*, comb. nov., in bat hibernacula of Eastern North America. Fungal Biol..

[76] Blehert D.S., Hicks A.C., Behr M., Meteyer C.U., Berlowski-Zier B.M., Buckles E.L., Coleman J.T., Darling S.R., Gargas A., Niver R. (2009). Bat white-nose syndrome: An emerging fungal pathogen?. Science.

[77] Drees K.P., Lorch J.M., Puechmaille S.J., Parise K.L., Wibbelt G., Hoyt J.R., Sun K., Jargalsaikhan A., Dalannast M., Palmer J.M. (2017). Phylogenetics of a fungal invasion: Origins and widespread dispersal of White-Nose Syndrome. mBio.

[78] Meletiadis J., Meis J.F.G.M., Mouton J.W., Verweij P.E. (2001). Analysis of growth characteristics of filamentous fungi in different nutrient media. J. Clin. Microbiol..

[79] Littman M.L. (1947). A culture medium for the primary isolation of fungi. Science.

[80] Ogórek R., Kalinowska K., Pląskowska E., Baran E., Moszczyńska E. (2011). Zanieczyszczenia powietrza grzybami na różnych podłożach hodowlanych w wybranych pomieszczeniach kliniki dermatologicznej. Część I/Mycological air pollutions on different culture mediums in selected rooms of dermatology department. (Part I). Mikol. Lek..

[81] Eisen J.A. (2007). Environmental Shotgun Sequencing: Its Potential and Challenges for Studying the Hidden World of Microbes. PLoS Biol..

[82] Wang W., Ma X., Ma Y., Maoa L., Wu F., Maa X., Ana L., Fenga H. (2010). Seasonal dynamics of airborne fungi in different caves of the Mogao Grottoes, Dunhuang, China. Int. Biodeterior. Biodegrad..

[83] Pilarek M. (1989). Polska Norma PN-89/Z-04111/03. Ochrona Czystości Powietrza. Badania Mikrobiologiczne. Oznaczanie Liczby Grzybów Mikroskopowych w Powietrzu Atmosferycznym (Imisja) Przy Pobieraniu Próbek Metodą Aspiracyjną i Sedymentacyjną/Polish Norm PN-89/Z-04111/03. Determination of the Number of Bacteria in the Atmospheric Air by aspiration and Sedimentation Sampling.

[84] WHO (1988). Indoor Air Quality: Biological Contaminants Report on a WHO Meeting, Rautavaara, 29 August–2 September 1988.

[85] Schwab C.J., Straus D.C. (2004). The roles of *Penicillium* and *Aspergillus* in Sick Building Syndrome. Adv. Appl. Microbiol..

[86] Sorrenti V., Di Giacomo C., Acquaviva R., Barbagallo I., Bognanno M., Galvano F. (2013). Toxicity of ochratoxin A and its modulation by antioxidants: A review. Toxins.

[87] Egbuta M.A., Mwanza M., Babalola O.O. (2017). Health risks associated with exposure to filamentous fungi. Int. J. Environ. Res. Public Health.

[88] Frisvad J.C., Thrane U., Samson R.A., Hoekstra E.S., Frisvad J.C., Filtenborg O. (2002). Mycotoxin production by commonfilamentous fungi. Introduction to Food- and Air Borne Fungi.

[89] Talcott P.A., Peterson M.E., Talcott P.A. (2013). Mycotoxins. Small Animal Toxicolo.

[90] Frisvad J.C., Filtenborg O. (1989). Terverticillate penicillia: Chemo-taxonomy and mycotoxin production. Mycologia.

[91] Mahomed K., Mlisana K. (2016). *Penicillium* species: Is it a contaminant or pathogen? Delayed diagnosis in a case of pneumonia caused by *Penicillium chrysogenum* in a systemic lupus erythematosis patient. Int. J. Trop. Med. Publ. Health.

[92] Geltner C., Lass-Flör C., Bonatti H., Müller L., Stelzmüller I. (2013). Invasive pulmonary mycosis due to *Penicillium chrysogenum*: A new invasive pathogen. Transplantation.

[93] Latgé J.-P., Chamilos G. (2019). *Aspergillus fumigatus* and Aspergillosis in 2019. Clin. Microb. Rev..

[94] Segal B.H. (2009). Aspergillosis. N. Engl. J. Med..

[95] Rhodes J.C., Jensen H.E., Nillius A.M. (1992). *Aspergillus* and aspergillosis. J. Med. Vet. Mycol..

[96] Kredics L., Varga J., Kocsubé S., Rajaraman R., Raghavan A., Dóczi I., Bhaskar M., Németh T.M., Antal Z., Venkatapathy N. (2009). Infectious keratitis caused by *Aspergillus tubingensis*. Cornea.

[97] Jarros I.C., Veiga F.F., Corrêa J.L., Barros I.L.E., Gadelha M.C., Voidaleski M.F., Pieralisi N., Pedroso R.B., Vicente V.A., Negri M. (2020). Microbiological and virulence aspects of *Rhodotorula mucilaginosa*. EXCLI J..

[98] Scholtz H.D., Meyer L. (1965). *Mortierella polycephala* as a cause of pulmonary mycosis in cattle. Berl. Muench Tieraerztl. Wochenschr..

[99] Kantarcioǧlu A.S., Boekhout T., De Hoog G.S., Theelen B., Yücel A., Ekmekci T., Fries B., Ikeda R., Koslu A., Altas K. (2007). Subcutaneous cryptococcosis due to *Cryptococcus diffluens* in a patient with sporotrichoid lesions case report, features of the case isolate and in vitro antifungal susceptibilities. Med. Mycol..

[100] Rapiejko P., Lipiec A., Wojdas A., Jurkiewicz D. (2004). Threshold pollen concentration necessary to evoke allergic symptoms. Int. Rev. Allergol. Clin. Immunol..

[101] Matsumoto T., Ajello L., Matsuda T., Szaniszlo P.J., Walsh T.J. (1994). Developments in hyalohyphomycosis and phaeohyphomycosis. Med. Mycol..

[102] Shahan T.A., Sorenson W.G., Paulauskis J.D., Morey R., Lewis D.M. (1998). Concentration- and time-dependent upregulation and release of the cytokines MIP-2, KC, TNF, and MIP-1 α in Rat alveolar macrophages by fungal spores implicated in airway inflammation. Am. J. Respir. Cell Mol. Bio..

